# Microvascularization of the Vocal Folds: Molecular Architecture, Functional Insights, and Personalized Research Perspectives

**DOI:** 10.3390/jpm15070293

**Published:** 2025-07-07

**Authors:** Roxana-Andreea Popa, Cosmin-Gabriel Popa, Delia Hînganu, Marius Valeriu Hînganu

**Affiliations:** Department of Morpho-Functional Sciences I, Faculty of Medicine, “Grigore T. Popa” University of Medicine and Pharmacy, 700115 Iasi, Romania; roxana.popa@umfiasi.ro (R.-A.P.); cosmin-gabriel.popa@umfiasi.ro (C.-G.P.); marius.hinganu@umfiasi.ro (M.V.H.)

**Keywords:** microvascularization, vocal folds, Reinke’s edema, capillary permeability, endothelial cells, pericytes, personalized vascular signaling

## Abstract

**Introduction**: The vascular architecture of the vocal folds plays a critical role in sustaining the dynamic demands of phonation. Disruptions in this microvascular system are linked to various pathological conditions, including Reinke’s edema, hemorrhage, and laryngeal carcinoma. This review explores the structural and functional components of vocal fold microvascularization, with emphasis on pericytes, endothelial interactions, and neurovascular regulation. **Materials and Methods**: A systematic review of the literature was conducted using databases such as PubMed, Scopus, Web of Science, and Embase. Keywords included “pericytes”, “Reinke’s edema”, and “vocal fold microvascularization”. Selected studies were peer-reviewed and met criteria for methodological quality and relevance to laryngeal microvascular physiology and pathology. **Results**: The vocal fold vasculature is organized in a parallel, tree-like pattern with distinct arterioles, capillaries, and venules. Capillaries dominate the superficial lamina propria, while transitional vessels connect to deeper arterioles surrounded by smooth muscle. Pericytes, present from birth, form tight associations with endothelial cells and contribute to capillary stability, vessel remodeling, and mechanical protection during vibration. Their thick cytoplasmic processes suggest a unique adaptation to the biomechanical stress of phonation. Arteriovenous anastomoses regulate perfusion by shunting blood according to functional demand. Furthermore, neurovascular control is mediated by noradrenergic fibers and neuropeptides such as VIP and CGRP, modulating vascular tone and glandular secretion. The limited lymphatic presence in the vocal fold mucosa contributes to edema accumulation while also restricting carcinoma spread, offering both therapeutic challenges and advantages. **Conclusions**: A deeper understanding of vocal fold microvascularization enhances clinical approaches to voice disorders and laryngeal disease, offering new perspectives for targeted therapies and regenerative strategies.

## 1. Background

The investigation techniques for studying the vascularization of the larynx have evolved significantly over time, driven by advancements in medical imaging, microscopy, and anatomical research methods.

Early methods primarily relied on anatomical dissections and observations of cadaveric specimens to understand the macroscopic vascular anatomy of the larynx. Pioneering anatomists such as Andreas Vesalius made significant contributions to our early understanding of laryngeal vascularization through meticulous dissections in the 16th century [1].

In the 19th century, the development of microscopy allowed for more detailed examination of laryngeal blood vessels at the microscopic level. Histological staining techniques enabled researchers to visualize the intricate network of capillaries, arterioles, and venules within the laryngeal mucosa [2].

With the advent of radiographic imaging in the 20th century, techniques such as angiography became valuable tools for studying the vascularization of the larynx in living patients. Angiography provided detailed images of the arterial supply to the larynx and helped identify pathological conditions such as vascular tumors or malformations [3].

In more recent decades, non-invasive imaging modalities such as computed tomography (CT) and magnetic resonance imaging (MRI) have revolutionized the study of laryngeal vascularization. These techniques allow for three-dimensional visualization of laryngeal anatomy and provide in-depth information about vascular structures and their relationships with surrounding tissues [4,5,6].

Furthermore, advanced endoscopic imaging techniques, including narrow-band imaging (NBI) and contact endoscopy, offer real-time visualization of laryngeal blood vessels during endoscopic procedures. These techniques enable clinicians to assess vascular patterns and identify abnormalities such as vascular lesions or tumors [7].

Overall, the history of investigation techniques for studying the vascularization of the larynx reflects a progression from anatomical dissections to advanced imaging modalities, each contributing to our understanding of laryngeal vascular anatomy and pathology.

### Purpose of the Study: The Importance and Complexity of Vascularization in the Vocal Apparatus

The purpose of this study is to investigate the intricate vascular architecture of the vocal apparatus, with a particular emphasis on the microvascularization of the vocal folds and the pivotal roles played by capillaries and pericytes. This vascular network is highly specialized to sustain the mechanical demands associated with phonation, particularly the intense and repetitive vibrations of the vocal folds, ensuring consistent perfusion even under substantial physical stress. The clinical significance of this vascular system is evident in pathologies such as Reinke’s edema and laryngeal carcinoma, where alterations in vascularization influence both disease progression and therapeutic response. Furthermore, this study highlights the dynamic interplay between pericytes and endothelial cells, which is essential for the development, stabilization, and remodeling of microvascular structures.

Recent research also points to the regulatory role of autonomic neurotransmitters—particularly noradrenaline—in modulating laryngeal vascular tone. Adrenergic fibers, through their distribution around laryngeal arteries, contribute to the control of regional blood flow, underscoring the neurovascular integration crucial to the functional integrity of the vocal mechanism. Understanding the mechanisms of angiogenesis and vascular morphogenesis within this context is fundamental for advancing regenerative strategies and targeted treatments in laryngeal pathology.

## 2. Materials and Methods

This historical review examines the development of knowledge regarding the microvascularization of the vocal folds, with a particular focus on Reinke’s edema, capillary permeability, endothelial cells, pericytes, and vascular signaling. The study is based on an extensive literature search conducted in major scientific databases, including PubMed (National Library of Medicine), Web of Science (Clarivate Analytics), Scopus (Elsevier), Embase (Elsevier), Cochrane Library, and Google Scholar, ensuring a comprehensive collection of peer-reviewed articles relevant to the topic.

**Figure 1:**
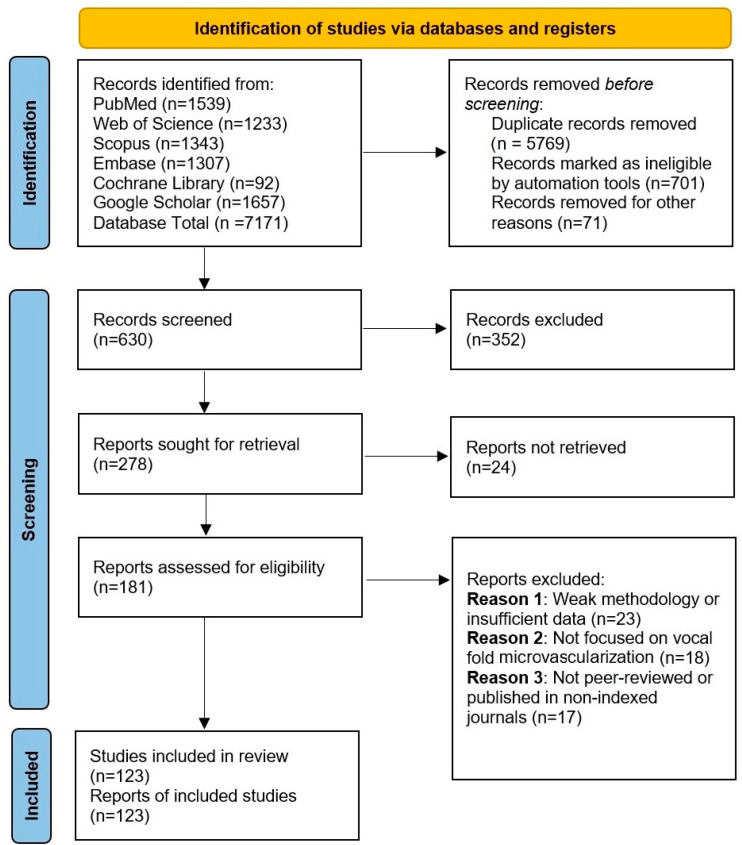
PRISMA flow diagram illustrating the study selection process for the systematic review. Initially, 7171 records were identified through database searching, of which 5769 duplicates were removed. After screening titles and abstracts, 352 records were excluded for not meeting the eligibility criteria. A total of 181 full-text articles were assessed for eligibility, with 58 excluded due to methodological quality concerns, resulting in 123 studies included in the final analysis.

The search strategy involved the use of specific keywords, including microvascularization, vocal folds, Reinke’s edema, capillary permeability, endothelial cells, pericytes, and vascular signaling. Boolean operators (AND, OR, NOT) were applied to refine the search results, along with filters to limit studies based on publication date, relevance, and peer-review status. Articles were selected based on their contribution to the historical understanding of vocal fold microvascularization and its pathophysiological implications. The screening and inclusion of studies are summarized in Figure 1.

The inclusion criteria encompassed peer-reviewed articles, systematic reviews, and historical perspectives published in indexed journals that provided significant insights into the microvascular physiology of the vocal folds. Studies with weak methodologies, non-peer-reviewed sources, or those lacking verifiable data were excluded.

The selected articles were analyzed chronologically to trace the progression of research on vocal fold vascularization, highlighting key discoveries and advancements. The findings were contextualized within the broader framework of microvascular biology, with an emphasis on the interactions between endothelial cells and pericytes and the role of vascular signaling in maintaining vocal fold function and pathology. This methodological approach ensures a rigorous synthesis of historical and contemporary perspectives on the subject.

## 3. Results

### 3.1. Personalized Approach on Embryonic Blood Vessel Formation: From Vasculogenesis to Angiogenesis

The initial stages of blood vessel development emerge in the yolk sac, coinciding with neural plate and somite formation. Within the splanchnic mesoderm, mesenchymal cells aggregate to form blood islands, where centrally located cells differentiate into embryonic hemoblasts, while peripheral cells give rise to endothelial cells. The fusion of adjacent vesicles leads to the formation of vascular channels, which subsequently extend toward the embryo through directed branching and elongation [8].

According to the literature, all embryonic blood vessels originate from extraembryonic precursors that expand through continuous budding, branching, and elongation, a process known as angiogenesis [9]. This mechanism closely parallels tumor vascularization [9,10] and wound-induced neovascularization [11]. However, subsequent studies identified intraembryonic endothelial vesicles forming independently within the splanchnic mesoderm. Unlike blood islands, these vesicles do not originate as mesenchymal aggregates and, with few exceptions [12], are not associated with blood cell precursor formation. These differences have led some researchers to propose that intraembryonic endothelial vesicles may instead represent transient extensions of vascular structures originating externally [13]. New blood vessels form through two primary processes: vasculogenesis and angiogenesis. Vasculogenesis involves the de novo assembly of endothelial cells into vessels, as seen in the formation of the dorsal aortae, aortic arches, and cardinal veins [14,15]. Some networks initially develop as capillary plexuses, which later remodel into hierarchical structures to meet tissue demands, such as the perineural plexus and the capillary plexus of the yolk sac. The yolk sac plexus connects the blood islands, the first site of blood formation, to the embryo [16,17]. In quail, endothelial cells from blood islands sprout and interconnect, while in mice, primitive blood cells initially associate loosely with angioblasts before endothelial differentiation ensheathes them [18,19]. Intraembryonic vessels, however, form independently through angioblast coalescence. Although vasculogenesis is essential in early vessel formation, the precise role of angiogenesis in these early stages remains unclear, requiring further investigation. However, as development progresses, angiogenesis plays a progressively dominant role in vascular formation.

**Figure 2:**
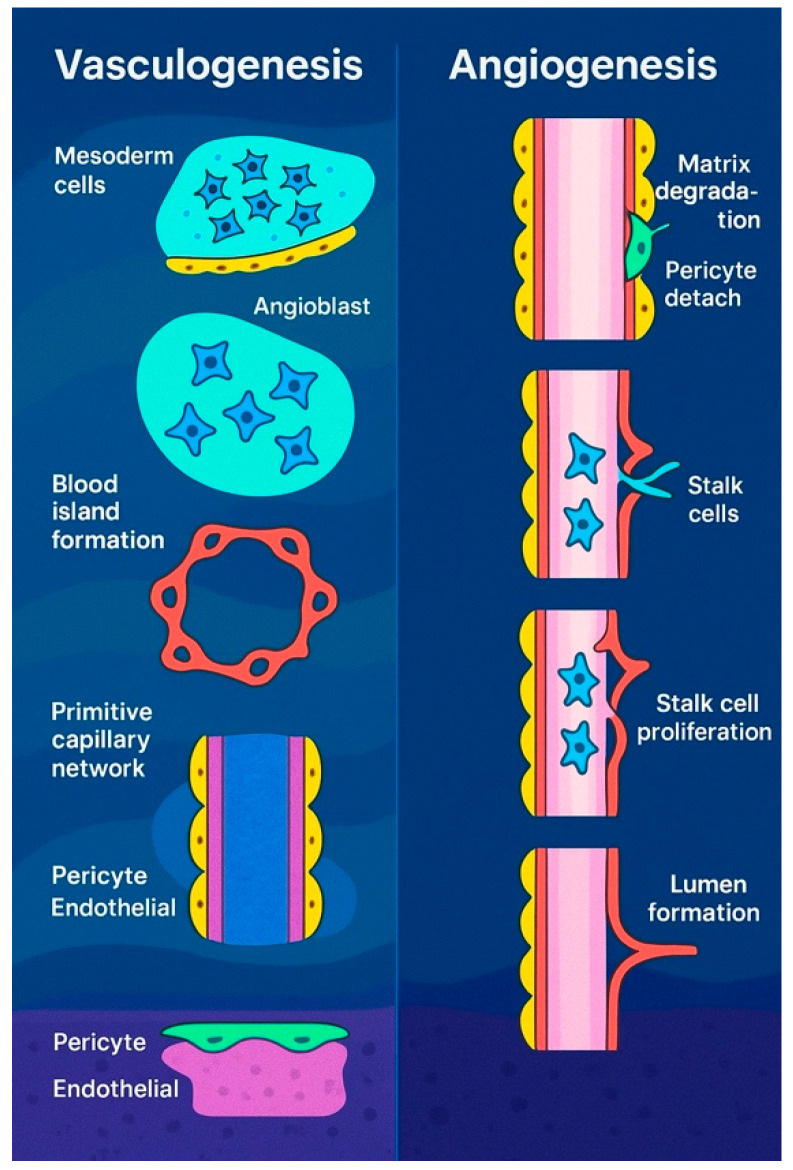
Vasculogenesis in embryos (**left**) refers to the formation of new blood vessels from primitive mesodermal cells. Signals from nearby endodermal cells stimulate these precursor cells to become angioblasts, which cluster to form blood islands. These islands then merge to create a primitive vascular network that later develops into the arteriovenous system. In contrast, angiogenesis (**right**) is the process of generating new blood vessels from existing ones. This begins with the breakdown of the extracellular matrix, the detachment of pericytes, and the emergence of tip cells. These tip endothelial cells, which do not divide, migrate toward sources of proangiogenic signals and are followed by proliferating stalk cells.

Beyond vasculogenesis, new vessels can form by sprouting from pre-existing vasculature, a process essential for the development of various structures, including the intersegmental vessels, retina, limbs, and central nervous system (CNS) [20,21]. This mechanism also plays a role in hypoxia response, tumor growth, and wound healing. First observed in the 1800s [22], sprouting angiogenesis (Figure 2) involves five key steps: initiation by pro-angiogenic factors such as VEGF and NOTCH signaling, specification of endothelial tip cells leading the sprout, formation of proliferative stalk cells regulated via NOTCH lateral inhibition [23], vessel outgrowth directed by VEGF gradients [24], and eventual fusion with neighboring vessels, potentially guided by macrophages [25,26]. Though VEGF is a primary mediator, factors like FGF2 also contribute [27]. While macrophages aid angiogenesis in repair and tumor interactions [28], their exact role in vessel anastomosis remains unclear.

### 3.2. Normal Microvascular Structure of the Vocal Folds

The microvascular system of the human vocal folds primarily consists of capillaries within the superficial lamina propria, along with smaller arteries, veins, arterioles, and venules in the deeper layers. Direct anastomoses exist between arterioles and venules, ensuring efficient blood circulation [29]. The vascular network of the lamina propria remains distinct from the underlying muscle layer, with only a limited number of blood vessels extending from the vocalis muscle into the lamina propria.

Under normal conditions, vessels on the ventricular side of the central vocal fold region follow a mildly undulating, tree-like branching pattern [30]. The middle portion of the vocal folds receives blood supply from both anterior and posterior directions, with the vessel diameter decreasing toward the center. Transverse vessels, when present, are sparse and narrow. In the muscle layer, blood vessels enter from the deeper regions and remain separate from those in the lamina propria of the mucosa. At the mid-point of the vocal fold, particularly on the inferior surface, a reticulated vascular network is present, with occasional direct arteriovenous anastomoses [31].

### 3.3. The Microarchitecture of Blood Vessels Within the Mucosa of the Vocal Fold

Within the mucosa at the edge of the vocal fold, the vascular structure predominantly comprises small vessels—arterioles, capillaries, and venules—aligned parallel to the fold’s edge. The superficial layer of the lamina propria, also referred to as Reinke’s space, is primarily composed of capillaries [32]. This layer contains few blood capillaries, rare seromucinous glands, and no lymphatic vessels. The absence of lymphatic drainage and reduced vascularity contribute to the confinement of carcinomas in this region, resulting in more favorable outcomes following surgical or radiotherapeutic treatment. Furthermore, impaired lymphatic drainage facilitates the accumulation of edema, promoting the development of nodular lesions or polyps [33].

Arterioles within the vocal fold mucosa are small, with diameters typically ranging from 300 μm to less than 50 μm [34]. These vessels are enveloped entirely by smooth muscle cells. Terminal arterioles exhibit a brief transitional zone characterized by scattered smooth muscle cells, eventually giving rise to arterial capillaries. These capillaries serve as an intermediate form, morphologically bridging smooth muscle cells and pericytes before transitioning fully into true capillaries.

Capillary walls consist of endothelial cells, basal lamina, and a sparse reticular fiber network. Their diameter generally ranges between 8 and 12 μm, which permits the unimpeded passage of cellular blood components. The endothelial lining is mostly smooth; however, overlapping cell margins and marginal folds are often noted. Some endothelial cells display circular fenestrations (60–70 nm) that are occluded by thin pore diaphragms [34].

Pericytes are frequently observed surrounding capillaries in the vocal fold mucosa [35]. Each pericyte features a fusiform or polygonal cell body and cytoplasmic processes—short circumferential and long longitudinal projections—aligned parallel to the capillary axis. These pericytes typically measure 5–10 μm × 15–30 μm, with cell bodies around 5–10 μm × 10–15 μm. The processes often display a fingerlike or clawlike morphology, grasping the capillary surface. A dense meshwork of cytoplasmic filaments and organelles, such as rough endoplasmic reticulum, mitochondria, and ribosomes, is present, especially within these processes. These filaments form dense bodies and contribute to the structural integrity of the capillary wall.

Pericyte cell bodies are typically located 300–500 nm from the endothelial cells, whereas their processes are in direct contact, sharing a common basement membrane and forming tight junctions at their distal ends, as seen in scanning electron microscopy (SEM). This arrangement is also evident in the newborn vocal fold mucosa, where the capillary architecture and pericyte positioning mirror those observed in adults, indicating the early establishment of vascular support mechanisms [36].

### 3.4. Functional Role of the Vascular Network in the Human Vocal Fold Mucosa

Small vessels enter the vocal fold edge from the anterior and posterior ends of the membranous vocal fold, running in parallel with the fold’s margin. These vessels remain functionally and anatomically distinct from those in the superior and inferior mucosal layers and from the vascularization of the vocalis muscle. This specialized arrangement is evolutionarily optimized to accommodate and support the dynamic mechanical demands of phonation, ensuring stability during vibration while minimizing circulatory disruption [37].

A key structural feature is the presence of direct arteriovenous anastomoses. When these structures contract, blood is routed through the capillary bed; when relaxed, blood bypasses the capillaries and flows directly into venules [36]. These anastomoses play a crucial role in regulating regional blood supply based on functional demand.

Arterioles act as primary regulators of vascular resistance and, by extension, systemic blood pressure. Capillaries are the principal sites for exchange between blood and surrounding tissue, while venules contribute to both exchange and inflammatory processes [38]. Collectively, this specialized vascular network architecture allows the mucosa of the vocal fold to resist hypoxia and accommodate the cyclical mechanical strain imposed by phonation.

### 3.5. Functional Importance of Capillary Pericytes in the Human Vocal Fold Mucosa

Pericytes have been extensively studied in various tissues, including through silver staining by Zimmermann [39] and electron microscopy in multiple organs [40]. Their number and morphology are tissue-specific and correlate with the density of the local capillary network [41]. Their shape and distribution are thought to reflect functional requirements.

Although their exact physiological functions remain partially understood, pericytes are thought to contribute to capillary contraction, structural support, cellular differentiation, sensory regulation, and synthetic activity. The presence of cytoplasmic filaments in pericytes suggests a contractile function, allowing them to modulate capillary diameter and microvascular flow [42,43,44,45]. Additionally, pericytes play a pivotal role in angiogenesis by stabilizing and guiding endothelial cells and facilitating blood vessel maturation [45].

In the vocal fold mucosa, numerous pericytes encircle both arterial and venous capillaries [31,35]. Morphologically similar to those found in other organs, these pericytes are distinguished by their relatively thick processes. These structural characteristics enable them to form tight junctions with endothelial cells and firmly envelop the vessels, providing robust support and protection against mechanical stress induced by vocal fold vibration.

The convergence of cytoplasmic filaments into dense bodies further enhances their mechanical role, making capillaries more resistant to rupture during phonation. Notably, blood flow within the mucosa decreases during phonation but increases post-phonation, underscoring the regulatory role that pericytes may have in maintaining capillary integrity and function during cyclical stress [46].

Moreover, pericytes contribute to tissue regeneration and revascularization following injury, playing a key role in angiogenesis. Their presence in the vocal fold mucosa from birth [47] indicates a pre-established structural mechanism ready to support vascular integrity immediately after birth.

### 3.6. Key Mechanisms in Blood Vessel Formation: Endothelial Cell–Pericyte Interactions and Vascular Morphogenesis

Understanding blood vessel formation and maturation is crucial for addressing diseases, cancers, tissue bioengineering, and regenerative medicine [48,49]. While progress has been made in vasculogenesis and angiogenesis, much remains unknown about the cell biology of key vascular cell types, such as endothelial cells (ECs), pericytes, and vascular smooth muscle cells, as well as the signaling processes that regulate tube formation, specialization, and vessel maturation (Table 1). Blood vessel formation, including vasculogenesis, angiogenesis, and pathologic neovascularization, involves complex interactions between ECs, mural cells (e.g., pericytes), parenchymal cells, and the extracellular matrix (ECM) [50]. These interactions are essential for morphogenic events such as tube formation, polarization, and the correct positioning of mural cells along EC tubes. EC–pericyte interactions, especially in capillaries, are crucial for tissue oxygenation and nutrient delivery, and abnormalities in these interactions are observed in diseases like diabetes and cancer. A deeper understanding of these processes is vital for normal development, disease treatment, and tissue engineering applications. Critical steps in EC tubulogenesis include ECM degradation, which creates tunnels for EC tube formation and pericyte recruitment, contributing to the remodeling of the vascular structure [51,52,53].

Pericytes are crucial regulators of endothelial cell (EC) tubulogenesis, tube remodeling, and vessel maturation [93,94]. Their recruitment leads to the formation of more elongated, branched, and narrower tubes compared to EC tubes without pericytes under similar conditions (Table 2). Pericytes control vascular morphogenesis through cell–cell interactions and by regulating basement membrane deposition, which influences signaling pathways. These signals are mediated through altered integrin signaling and integrin/growth factor co-signaling, with factors like BMP-4 potentially altering signals when a vascular basement membrane forms [95,96,97]. In the context of diseases such as diabetes and cancer, abnormalities in EC–pericyte interactions are a known issue. Studies indicate that pericytes stabilize EC-lined tubes and prevent regression of vascular networks [98,99,100]. Pericyte-derived TIMP-3 plays a key role in preventing vascular regression by inhibiting MMPs from ECs, which would otherwise promote ECM degradation and vessel breakdown [93,101,102]. The suppression of TIMP-3 disrupts tube diameter and collagen type IV assembly, demonstrating its importance in vessel integrity. Additionally, reduced pericyte coverage in the central nervous system impairs the blood–brain barrier [71]. In conclusion, the disruption of pericyte recruitment, retention, or survival leads to unstable vasculature and altered EC biology, highlighting the complexity of vascular morphogenesis and maturation.

A critical point is that both in vitro and in vivo experimental approaches are essential for addressing these questions and ultimately developing a comprehensive understanding of these processes.

Various in vivo models are currently being utilized to investigate the molecular mechanisms underlying vascular morphogenesis, maturation, and stabilization, including models in mice, zebrafish, and avian species. A significant advancement in the utility of in vitro approaches to address these questions was the establishment of three-dimensional (3D) matrix vascular morphogenic models, which employ either isolated human endothelial cells or tissue fragments such as the rodent aorta [103,104,105].

These 3D matrix systems are particularly valuable for investigating vascular morphogenesis, as processes such as tubulogenesis and angiogenic sprouting occur exclusively within a 3D matrix environment [106,107]. The implementation of 3D matrix assay systems has proven to be of considerable importance due to their ability to accurately recapitulate in vivo vascular network formation, thereby enhancing the understanding of the factors and signaling events required for these processes [108,109].

Recent studies have developed and routinely utilize a serum-free, factor-defined three-dimensional (3D) matrix system that incorporates both collagen type I and fibrin matrices [110,111]. This system has enabled the identification of hematopoietic stem cell cytokines that promote human endothelial cell (EC) tubulogenesis in conjunction with fibroblast growth factor (FGF)-2, specifically the combination of stem cell factor (SCF), stromal-derived factor-1α (SDF-1α), and interleukin-3 (IL-3). Additionally, Bowers et al. have demonstrated that this system facilitates the analysis of EC–pericyte interactions, wherein pericytes recruit to developing EC tubes, participate in basement membrane matrix deposition, and travel within the tunnels created by ECs during EC tubulogenesis [110,111,112]. As shown, a variety of techniques are employed to analyze cell–cell and cell–extracellular matrix (ECM) relationships during vasculogenic tube assembly, including growth factor signaling, protein kinase cascades, GTPase regulation, and integrin signaling. A novel technique described by Bowers et al. is the combination of their defined 3D matrix system with a gene-inducible lentiviral system, allowing for the regulation of a gene of interest by ECs or pericytes (following the addition of doxycycline) at specific time points during tube formation or maturation [48].

### 3.7. The Role of Neuropeptides in the Regulation of Laryngeal Vascularization

Studies on the canine larynx have demonstrated that adrenergic neurotransmission plays a key role in the regulation of laryngeal vascularization. Using electron microscopy and fluorescence histochemistry with the 5-hydroxydopamine (5-OHDA) method, researchers have identified adrenergic nerve terminals in proximity to vascular structures, particularly around the base of acini in the submucosa and adjacent to blood vessels within the intrinsic laryngeal muscles. These findings suggest a direct influence of sympathetic innervation on local blood flow dynamics.

To determine the origin and distribution of noradrenergic fibers within the laryngeal nerves, further analysis was conducted using the Falck–Hillarp fluorescence method following selective denervation of the superior and inferior laryngeal nerves, with preservation of the corresponding arteries and veins. It was found that noradrenergic fibers supplying the supraglottic and subglottic submucosal glands originate from the internal branch of the superior laryngeal nerve and the recurrent laryngeal nerve, respectively. The external branch of the superior laryngeal nerve innervates the cricothyroid muscle, while other intrinsic laryngeal muscles receive adrenergic input from the internal branch and recurrent nerve. These fibers primarily arise from the superior cervical ganglion, although some also derive from the middle cervical ganglion via the vagus nerve. Denervation studies confirmed that these are the exclusive sources of adrenergic input to the larynx [113,114].

Immunohistochemical detection of tyrosine hydroxylase (TH), a biosynthetic enzyme for catecholamines, provided further evidence of adrenergic involvement in laryngeal vascular regulation [114]. TH-positive fibers were frequently observed surrounding arteries in the laryngeal mucosa and within intrinsic muscles, underscoring their role in arterial vasomotor control. However, these fibers were rarely found around capillaries, suggesting that microvascular regulation at the capillary level may not be under direct adrenergic control. Regional distribution patterns also revealed higher densities of TH-positive fibers in the glottic and supraglottic mucosa, with notably fewer fibers present in the subglottic region. Additionally, in glandular regions, some TH-positive fibers were seen terminating near the basement membranes of glandular cells, implying a role not only in vascular modulation but also in the regulation of glandular secretion.

In conclusion, these findings highlight the critical function of adrenergic nerve fibers in modulating the vascularization of the canine larynx, particularly through control of arterial blood flow, with distinct anatomical and functional distribution across laryngeal regions.

In addition to noradrenaline and acetylcholine, non-noradrenergic, non-cholinergic (NANC) transmitters have been identified as important components of the autonomic nervous system (ANS). Among these, neuropeptides—peptides synthesized and secreted by neurons—function both as neurotransmitters and neuromodulators. Since the 1970s, various neuropeptides have been discovered not only in neurosecretory neurons of the hypothalamus but also in neurons distributed across multiple organs. These neuropeptides are often co-localized with classical neurotransmitters [115].

For instance, neuropeptide Y (NPY) is co-localized with noradrenaline in the peripheral sympathetic nervous system and is also associated with choline acetyltransferase (ChAT) and adenosine triphosphate (ATP). Similarly, vasoactive intestinal peptide (VIP) is co-released with acetylcholine from postganglionic fibers in the parasympathetic nervous system [115,116].

Recent studies have demonstrated the presence and specific roles of neuropeptides in the laryngeal nervous system. The following neuropeptides are among the most representative in the larynx:

- Neuropeptide Y (NPY): A 36-amino-acid peptide originally isolated from the porcine brain, with its molecular structure first elucidated by Tatemoto in 1982 [117]. NPY is co-localized with noradrenaline in sympathetic nerve terminals and is co-released with it. It is believed to play a significant role in angiogenesis during tissue development and repair processes [118,119].
- Vasoactive Intestinal Peptide (VIP): A 28-amino-acid peptide initially identified in porcine intestine in 1974. VIP is widely distributed in both the central and peripheral nervous systems and functions primarily in the relaxation of intestinal smooth muscles, dilation of peripheral blood vessels, and stimulation of salivary secretion [120].
- Calcitonin Gene-Related Peptide (CGRP): A 37-amino-acid peptide derived from alternative splicing of the calcitonin gene, first characterized in the 1980s. CGRP is extensively distributed throughout the central and peripheral nervous systems, where it plays key roles in vasodilation, pain transmission, and inflammatory processes [121].

Tanaka et al. (1995) provided a detailed immunohistochemical analysis of the distribution of VIP, NPY, and tyrosine hydroxylase (TH) in the larynx using electron microscopy [122]. VIP-positive fibers were observed surrounding the basal membrane and myoepithelial cells of the laryngeal glands. Some of these fibers made contact with the basal lamina, while others penetrated it, running intercellularly among adjacent glandular cells without forming synaptic contacts.

In contrast, fibers positive for NPY and TH were also found near the basal lamina but were comparatively less abundant than the VIP-positive fibers in this region.

## 4. Conclusions

Understanding the microvascular architecture of the vocal folds holds significant clinical relevance in otolaryngology and voice medicine. The intricate organization of capillaries, pericytes, and arteriovenous structures plays a pivotal role in maintaining tissue homeostasis under the mechanical stress of phonation. The disruption of vascular integrity is associated with pathologies such as Reinke’s edema, hemorrhage, and laryngeal neoplasia. Insights into pericyte–endothelial cell interactions and the neurovascular regulatory axis, particularly the influence of adrenergic and peptidergic innervation, offer potential therapeutic targets for managing voice disorders and improving surgical outcomes. Moreover, the unique vascular characteristics of the vocal fold mucosa—such as the lack of lymphatics and the specialized capillary network—contribute to both the containment of malignancies and the predisposition to edema formation. A detailed understanding of these mechanisms not only advances diagnostic precision through modern imaging but also informs regenerative strategies and pharmacologic interventions aimed at preserving or restoring vocal function. Importantly, these insights pave the way for personalized medicine approaches by enabling targeted diagnostics and therapies tailored to the individual vascular and neuroregulatory profiles of patients with vocal fold disorders.

## Figures and Tables

**Table 1:** Identified pericyte markers.

Marker	Associated Cell Types	References
PDGFR-β	Myofibroblasts, neurons and progenitors, mesenchymal cells, mesenchymal stem cells	[54,55,56,57]
PDGFR-α	Mesenchymal cells, neural stem cells/B cells	[58,59,60,61]
NG2	vSMC, adipocytes, neuronal progenitors, glial cells, developing bone, muscle, skin	[62,63,64,65,66,67]
Desmin	Skeletal muscle cells, cardiac smooth muscle cells, mesangial cells	[55,68,69,70]
α-SMA	vSMC, myofibroblasts	[71,72,73,74]
RGS5	vSMC	[75,76,77]
Endosialin	Myofibroblasts, fibroblasts, vSMC	[78,79,80]
CD73	Mesenchymal stem cells	[55]
CD13	vSMC, epithelial cells in the kidneys, tumor endothelial cells	[81,82,83]
CD146	Mesenchymal stem cells	[84]
CD105	Mesenchymal stem cells, endothelial cells, hematopoietic stem cells	[84,85,86,87]
CD44	Mesenchymal stem cells, lymphocytes, hematopoietic stem cells	[88,89]
ANGPT1	Hematopoietic progenitor cells, glioblastoma tumor cells, mast cells	[31,90]
VEGF-A	Tumor cells, macrophages	[91,92]

**Table 2:** Comparative functional properties of endothelial cells and pericytes during vascular development and maturation.

Functional Property	Endothelial Cells (ECs)	Pericytes
Tube Formation in 3D Collagen/Fibrin Matrices	ECs have the ability to form tubes in 3D collagen or fibrin matrices.	Pericytes do not form tubes in 3D matrices but invade as single cells.
Vascular Guidance Tunnel Creation	ECs create vascular guidance tunnels during tubulogenesis in 3D matrices, aided by ECM proteolysis, particularly through MT1-MMP activity.	Pericytes invade in response to ECs in a manner dependent on PDGF-BB; recruitment results in more elongated and narrow EC tubes.
Migration and Tube Assembly	ECs dramatically migrate within 3D matrices and co-assemble into tubes within vascular guidance tunnels.	Pericytes express very high levels of PDGFRβ and use this receptor to invade EC tubes and migrate along the tube abluminal surface.
Proliferation during Tube Formation	ECs exhibit minimal to no proliferation during tube formation in 3D matrices.	Pericytes proliferate in response to ECs in a manner dependent on PDGF-BB and HB-EGF in 3D matrices.
Co-assembly with Pericytes and Basement Membrane Formation	ECs co-assemble with pericytes to form capillary vessels and generate the vascular basement membrane.	Pericytes migrate along the EC tube abluminal surface within vascular guidance tunnels to facilitate basement membrane formation.
Response to Hematopoietic Cytokines	Human ECs form tubes and sprout in response to stem cell cytokines (SCF, IL-3, SDF-1α) and FGF-2 under serum-free, defined conditions.	Human EC–pericyte tube co-assembly with accompanying basement membrane formation occurs in 3D matrices in response to hematopoietic cytokines.

## Data Availability

Data are contained within the article.
